# Euros vs. Yuan: Comparing European and Chinese Fishing Access in West Africa

**DOI:** 10.1371/journal.pone.0118351

**Published:** 2015-03-20

**Authors:** Dyhia Belhabib, U. Rashid Sumaila, Vicky W. Y. Lam, Dirk Zeller, Philippe Le Billon, Elimane Abou Kane, Daniel Pauly

**Affiliations:** 1 *Sea Around Us*, Fisheries Centre, University of British Columbia, 2202 Main Mall, Vancouver, Canada; 2 Fisheries Economics Research Unit, Fisheries Centre, University of British Columbia, 2202 Main Mall, Vancouver, Canada; 3 Department of Geography, University of British Columbia, 1984 West Mall, Vancouver, BC, Canada; 4 Oceanographic and Fisheries Mauritanian Research Institute, BP. 22 Nouadhibou, Mauritania; Aristotle University of Thessaloniki, GREECE

## Abstract

We compare the performance of European Union (EU) and Chinese fisheries access agreements with West African countries in terms of illegal and unreported fishing, economic equity, and patterns of exploitation. Bottom-up re-estimations of catch reveal that the EU (1.6 million t•year^-1^) and China (2.3 million t•year^-1^) report only 29% and 8%, respectively, of their estimated total catches (including estimated discards whenever possible) from West African countries between 2000 and 2010. EU catches are declining, while Chinese catches are increasing and are yet to reach the historic maximum level of EU catches (3 million t•year^-1^ on average in the 1970s-1980s). The monetary value of EU fishing agreements, correlated in theory with reported catches, is straightforward to access, in contrast to Chinese agreements. However, once quantified, the value of Chinese agreements is readily traceable within the African economy through the different projects they directly cover, in contrast to the funds disbursed [to host governments] by the EU. Overall, China provides resources equivalent to about 4% of the ex-vessel value [value at landing] of the catch taken by Chinese distant-water fleets from West African waters, while the EU pays 8%. We address the difficulties of separating fees directly related to fishing from other economic or political motivations for Chinese fees, which could introduce a bias to the present findings as this operation is not performed for EU access fees officially related to fishing. Our study reveals that the EU and China perform similarly in terms of illegal fishing, patterns of exploitation and sustainability of resource use, while under-reporting by the EU increases and that by China decreases. The EU agreements provide, in theory, room for improving scientific research, monitoring and surveillance, suggesting a better performance than for Chinese agreements, but the end-use of the EU funds are more difficult, and sometime impossible to ascertain.

## Introduction

Increasing seafood demand, particularly in Europe and Asia, and the depletion of local fish stocks contributed greatly to the growth of heavily subsidized distant-water fleets (DWF) from Europe and Northeast Asia, which have increased their effort in the ‘south’, i.e., to the developing world [1]. Thus, over 70% of the European Union (EU) seafood is now imported from outside EU waters, mostly from the developing world [2–5]. It is worth noting that the situation is similar for Japan [3] and likely also for China [6].

The lack of adequate financial and technical resources often limits proper monitoring, control and enforcement of industrial foreign fishing activities in the developing world, resulting in overfishing and under-reported and unsustainable catches [7–10]. Catch data by DWF, in instances where host countries’ monitoring and surveillance capacity is low, are rarely reliable [11,12]. This often leads to large gaps between reality and official statistics provided to the United Nations Food and Agriculture Organization (FAO).

Only a few developing countries exploit their offshore stocks themselves, due to a general lack of technical, financial and governance infrastructure to develop and maintain industrial fishing vessels. Ghana and Thailand are two exceptions that come to mind, see [13] and [14], respectively. This constrains most developing countries to selling the so-called ‘surplus’, i.e., the catch between the domestically inaccessible maximum sustainable yield and their domestic fisheries landings [15]. Indeed, under the 1980s United Nations’ Convention on Law of the Sea (UNCLOS), countries shall provide access to this surplus to other countries through diverse access agreements [16]. The host country, however, may prioritize the local economy and regional needs before granting fishing access to other countries; yet, this is rarely the case for economically weak countries struggling to obtain foreign currency and service their external debts [17]. Furthermore, many countries prefer short-term benefits through fisheries access agreements where fees are negotiated based on the income generated by the foreign fishing capacity rather than (and regardless of) a sustainable catch quota [8] which in many cases is not known. Thus, their governments fail to use potential maximum sustainable yields and realistic catch estimates to limit the number and size of foreign vessels that access their fishing grounds. Moreover, these governments underestimate the importance of domestic activities such as processing and marketing which further limits local development.

Another issue is the myth that “there are no data” to consider appropriate and sustainable levels of catches. While it is true that the governments of developing countries rarely have sound science-based fisheries policies, this is not really due to a “lack of data”. Rather, it is mostly due to the lack of (support for) scientists to make sense of available data on catch and fishing effort by various fleets, and of ancillary economic data. As an example, we will demonstrate that the data available on European and Chinese distant-water fisheries in West Africa are sufficient for inferences on their catch and its value, and for first, if tentative, comparisons between the fees that the EU and China are paying in return for the right to fish in West Africa, all these being items that are highly relevant to fisheries policy. The present study seeks to first estimate then compare fees paid under agreements by the EU and China for their access to West African fisheries while comparing their reporting practices, landed value and illegal fishing activities.

## Fishing opportunities off West Africa

### 1. Study area

**Fig 1:**
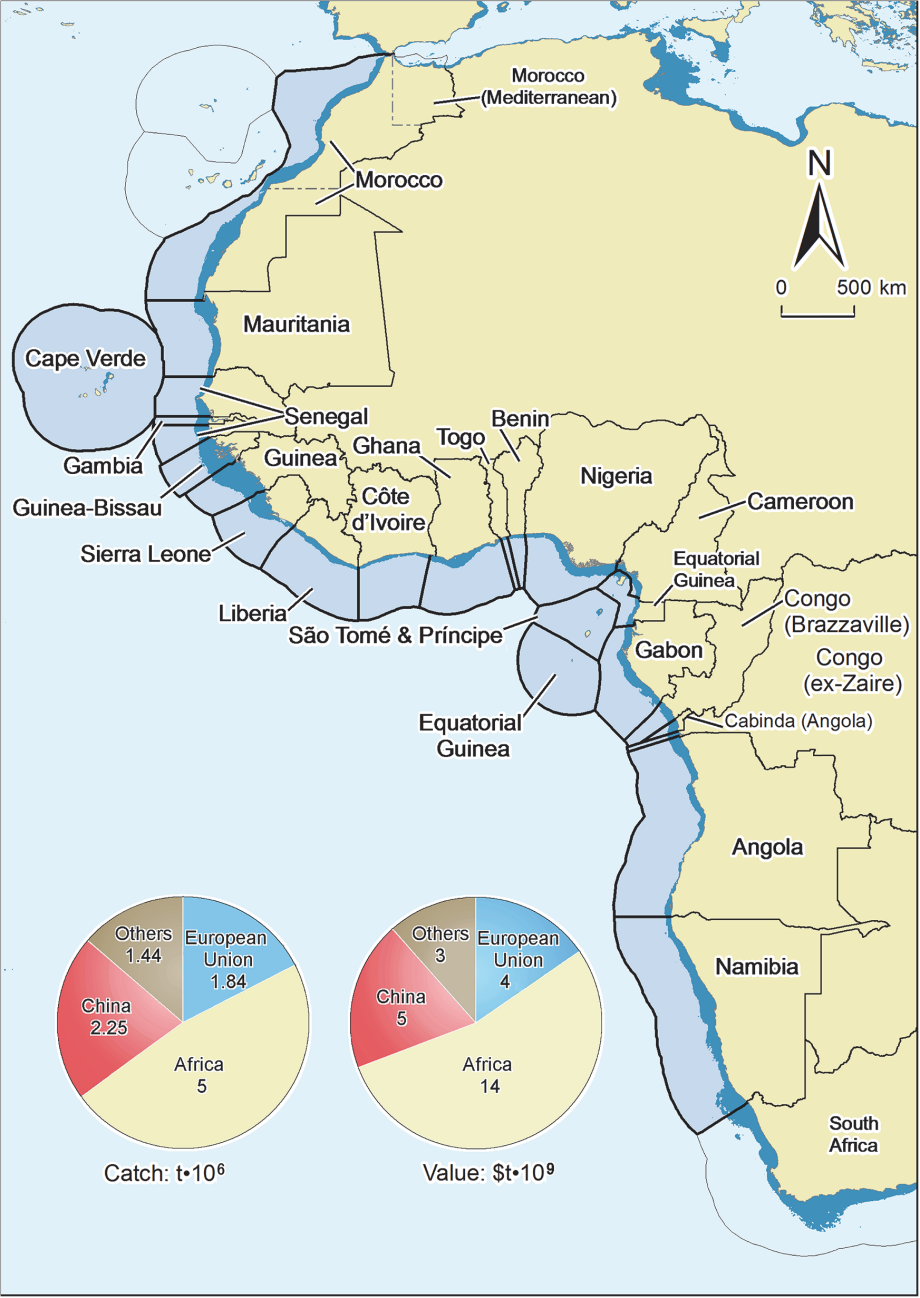
Exclusive Economic Zone waters of the West African countries considered here, also showing the 2000–2010 average annual catch and landed value of their marine fisheries, incl. distant-water fleets.

West Africa (Fig. 1) refers to the area between the Strait of Gibraltar (36° 8' N and 5° 21' W) and the extreme south of Namibia (17°15'S, 11°48'E), i.e., excluding South Africa. This area, encompassed within FAO statistical areas 34 (Eastern Central Atlantic) and 47 (South Eastern Atlantic) includes the following countries: Morocco (incl. Western Sahara), Mauritania, Cape Verde, Senegal, The Gambia, Guinea Bissau, Guinea, Sierra Leone, Liberia, Côte d’Ivoire, Ghana, Togo, Benin, Nigeria, Cameroon, Equatorial Guinea, Gabon, Sao Tome and Principe, Congo (Brazzaville), Congo (ex-Zaire), Angola and Namibia (Fig. 1). The foreign catch data taken from Namibian waters included only the EU component before the early 2000s.

### 2. Foreign access to offshore resources

In West Africa, high marine productivity induced by the Canary Current [18], Guinea Current [19] and Benguela Current upwelling systems [20,21], the inability of local fleets to access offshore resources, the depletion of fish stocks in other parts of the world, and increasing global fish demand are all drivers for foreign fishing [11,22–24]. Indeed, West Africa has been attractive to developed countries for some time through fishing agreements or illegal fishing [25,26].

Fishing agreements are here defined as the right to access living marine resources (here collectively defined as ‘fish’) within a host country's Exclusive Economic Zone (EEZ) in exchange for financial compensation. Legal access can be under bilateral or multilateral agreements between (a) the host government and an inter-government organisation like the European Union (EU), for example, the Fishing Partnership Agreements (FPAs), (b) the host government and DWF flag-country government, and c) a company and the host government, for example under (private) joint venture agreements (so-called ‘second generation agreements’), whose terms and conditions are often obscure [27]. Second generation agreements generally involve either the charter or the (temporary) transfer of fishing vessels to a third country, which in principle translates into reflagging industrial fleets to the host country. For example, most of the Mauritanian industrial fleet consists of Chinese-Mauritanian joint ventures, and the Senegalese industrial fleet consists largely of EU reflagged vessels [28,29]. Just like China, EU fleets are involved in a large number of joint ventures with, e.g., Guinea Bissau, Guinea, Sierra Leone, Gabon, Angola and Namibia in West Africa. The latest official estimate of landings and effort was available for 1997 when China landed 57,500 tonnes using around 53 vessels under the flags of West African countries [30]. The unavailability of more recent official data for China and the difficulty to retrace joint ventures by vessels from the EU illustrate an important issue of lack of transparency which is herein addressed.

Alder and Sumaila [26] estimated that fishing agreements to access West African fishing grounds increased following the global demand of fish, from 36 agreements in the 1960s to 242 by the 1980s (following the declaration of EEZs based on UNCLOS) and 302 agreements in the early 2000s. These numbers exclude the ‘domestic’ (offshore) fleets, which in most cases consist of reflagged European and Chinese vessels [28,31,32]. Indeed, more recently, figures show that industrial fishing vessels in the West African Sub-Region alone (encompassing 7 countries from Mauritania in the north to Sierra Leone in the south) reached over a thousand vessels, including 700 foreign vessels from the EU, China and South Korea [33]. One consequence of this is that fish caught in West Africa contributes about 25% of fish caught by European countries [26].

The political and economic bargaining power of most West African countries is low, and thus, the benefits generated from West African fisheries resources are often not equitably shared between West African host countries and DWF countries [7,8,34]. Also, most of these West African countries still lack the resources for monitoring and enforcing conditions underpinning these access agreements despite significant improvement notably in Morocco, Mauritania, Sierra Leone and Namibia. Terms and conditions accessed at http://eur-lex.europa.eu for West Africa usually (but not exclusively) lie in the offloading of catches in host country ports, reporting catches to host and home countries, paying for extra capacity, and finally paying a negotiated amount in exchange of access to the resources of West African countries. More commonly, however, the amount paid covers mostly the access to resources within Exclusive Economic Zones (EEZs) of West African countries, with no consideration [at least in reality] for the state of the resource, human rights or the governance level within the country. This is particularly true when these countries are heavily dependent on access payments for their foreign exchange [15], despite existing human rights issues [35,36], regardless of the state of already fully- or over-exploited resources, and/or under obscure conditions involving, e.g., corruption and blackmail [12].

Abundant and valuable resources and poor monitoring/enforcement as a result of problem-ridden governance provide perfect conditions for largely unregulated and often illegal foreign fishing off West Africa. Fishing access agreements allowed West African countries to capture part of the value of their fisheries resources, which were accessible to DWF countries for free prior to the declaration of their EEZs (beyond then established territorial waters). However, the financial compensation received, the economical returns on development, the coherence and even the legality of mainly the EU agreements were often questioned, as they are easily accessed. Yet, so far, only a few studies had measured the income generated under these or any other agreements [7,8,35,37,38]. France, Spain, Portugal, Greece and Italy are the main countries acting under public EU-Africa agreements. Other countries from Western, Northern and Eastern Europe, Asia, Flag of Convenience countries (FoC) and others also fish extensively in West Africa (see Table 1 for references).

**Table 1:** Average annual reconstructed and reported catches (2000–2010) by the EU and China from West African waters (t·10^3^).

Country	Reconstructed catch (t·10^3^)		Reference	Reported catch (t·10^3^)	
	EU	China		EU	China
Morocco	522.4	595.7	[77]	168.8	42.0
Mauritania	618.7	841.5	[32]	143.0	91.2
Senegal	85.7	42.2	[43,78]	23.8	0.9
Cape Verde	19.1	13.9	[54]	6.2	1.4
The Gambia	20.8	10.1	[79]	6.7	0.8
Guinea Bissau	73.4	88.5	[97]	21.8	7.5
Guinea	333.3	49.1	[9]	106.8	2.9
Sierra Leone	18.5	19.7	[80]	5.9	0.2
Liberia	31.3	23.3	[81]	4.7	0.8
Côte d’Ivoire	3.0	84.8	[82]	0.9	1.3
Ghana	1.4	11.9	[83]	0.0	1.2
Togo	11.0	17.1	[84]	3.6	0.5
Benin	1.2	2.3	[85]	1.2	0.4
Nigeria*	0.0	168.5	[86]	0.0	0.0
Cameroon	0.0	9.7	[98]	0.0	0.2
Equatorial Guinea	9.0	4.9	[87]	2.9	0.5
Sao Tome & Prin.	6.6	0.2	[88]	2.1	<0.1
Gabon	25.2	55.2	[89]	8.1	4.7
Congo (Brazz.)	0.0	23.5	[90]	0.0	2.1
Congo (ex-Zaire)	0.0	11.7	[91]	0.0	1.1
Angola	56.3	186.9	[92]	17.8	19.7
Namibia**	NA	NA	NA	NA	NA
Total	1,836.9	2,251.0		524.3	179.2

*No documented fishing operations by the EU;

** We could not retrace Chinese legal catches from Namibia since all foreign vessels operating in Namibia have to be flagged to Namibia. Having vessel information would have allowed however the identification of the beneficial ownership of vessels; evidence of reflagging to China between 2000 and 2010 was scarce and was not sufficient to estimates the legal catches of Chinese vessels reflagged to Namibia.

Growing Chinese engagement in Africa has drawn significant attention in Europe [39–41]. In the fisheries sector, increasing fishing effort by Europe and China on the same resources over time suggests increasing competition to secure access to West African fishing grounds [42]. In any rational economic market, competition to acquire a limited resource, i.e., in high demand, leads to higher prices for this resource, in this case, access to highly valuable fishing grounds. In West Africa, despite increasing competition between the EU and China (and others such as Korea and Eastern European countries whose involvement focuses mainly on northwest African countries), which should imply a high demand for West African resources (particularly those in the north), Europe pays very different compensation to, e.g., Morocco and Mauritania (see agreements at http://eur-lex.europa.eu), which provides evidence of assigning low value to fish that command high demand. On the other hand, the increasing scarcity of fish resources, driven and caused in part by illegal foreign fishing and the expansion of domestic small-scale fishing, creates a vicious circle where, driven by declining stocks, fishing is temporally and spatially expanding to offshore waters by the artisanal fleets [43] and near-shore waters by industrial vessels, including into waters frequently legally reserved for the exclusive use of domestic small-scale fishers [44].

### 3. Importance and impacts of fishing on local communities

In West Africa, fisheries (excluding access fees) account for over 20% of the primary sector [45]. Also the jobs provided by the fisheries sector allow people to purchase high calorie staples such as rice and wheat. Growing occurrence and increasing intensity of challenging climate events (e.g., cyclical droughts) drive populations to the coast in search for alternatives to agriculture and pastoralism, leading to increasing domestic fishing pressures [46–48]. In turn, fisheries are sensitive to climate change, particularly in Sub-Saharan Africa [49], and where food security is highly dependent on fish [50]. It is worth considering the conundrum that collapsing fisheries and low income from fishing may result in trapping coastal populations between failing agriculture as droughts occurrence and intensity increase and failing fisheries.

Overexploitation of fish stocks, the shift towards higher market value species and export subsidies (e.g., export tax credits) threaten the integrity of domestic food security in host countries [51,52]. Increasing exports to Europe limit the range of fish available for consumption by local populations to small pelagic species, whose abundance is strongly climate dependent [50]. This puts further pressure on already heavily exploited stocks (by foreign fleets), thus reducing fish supply at a regional and domestic level [53].

### 4. Value of West African fisheries to EU and Chinese Distant Water Fleets

For the purpose of analyzing fisheries policy and foreign trade, landed values of catches are highly relevant. Indeed, these values drive the behavior of the heavily subsidized DWFs. Subsidies are payments from public entities towards, e.g., the industry. These encompass fees paid for access to foreign fisheries defined under the capacity enhancing subsidies [4], which provide an important incentive for DWF to pursue their activities. The EU pays these in the form of bilateral access agreement fees which also involve the contribution of the fishing industry, while China usually makes payments in the form of government development aid. China is estimated to provide over $4.1 billion USD annually to its DWF globally, which is slightly lower than what the EU pays ($4.6 billion US annually) [4]. Compensation for West African fisheries would be expected to be low, and the landed value in the international market more important given increasing foreign fishing capacity. Moreover, local governments of West Africa are unable to enhance domestic food security or implement poverty reduction strategies, with increasing competition between DWFs and with unknown or under-reported catches. The compensation received by West African countries under access agreements is often opaque and the conditions under which these agreements are signed are often confidential, except for the EU FPAs. However, even FPAs (or simply ‘fishing agreements’ prior to 2004) were shown to be unbalanced in terms of past and present compensation value [38], and in terms of overcapacity and reporting [54,55]. The ‘value’ of the catch taken by foreign fleets is always taken to be only its ex-vessel value, without value-added contributions. Moreover, host countries’ lack of capacity to monitor DWF activities and enforce agreement conditions make it difficult for them to benefit from such agreement. The latter should be aimed at local development. This is the reason why host countries often insist on at least some of the catch of DWFs to be landed and processed locally. However, this issue is not dealt with here and requires further research. If foreign fleets under-report their catches, the financial compensation for access agreements or joint ventures will not reflect the actual landed value of foreign catches from West African resources. Here, we define the value of foreign catches as the value of total reported and unreported catches. Thus, the issue of unreported, and potentially illegal catches, is important when assessing the compensation received. Furthermore, it raises the question of accountability at an international level: who is to be held responsible for under-reporting and illegal fishing of these fleets?

### 5. Objective of the study

The focus of this study is to evaluate and compare the EU-West Africa and China-West Africa fishing agreements with regards to the compensation West African countries receive from the EU and China. As a first step, we investigate the reliability of fisheries catch data by countries of the EU and China, by comparing official catch data to more comprehensive literature-based reconstructed [56] fisheries catches by the EU and China. The overall landed value of Chinese and EU fisheries off West African countries is estimated. Then, given the negative European perception of China’s growing engagement with West African countries [39], a per-country analysis will attempt to compare how Europe, under the EU-West African FPAs performs relative to Chinese agreements in terms of a) illegal, unreported and unregulated (IUU) fishing; b) amount of compensation; and c) patterns of exploitation of resource use.

## Material and Methods

### 1. Catches of the EU and China

We extracted industrial foreign catch data estimated using a bottom-up catch reconstruction approach [56] for the EEZs of the West African countries listed above, excluding Cameroon between 1950–2010 and Namibia during the 2000s, whose industrial catches are considered domestic (see Table 1), and refer to these data as ‘reconstructed catches’. One basic assumption of this method is rejecting zero as a reasonable estimate for a fisheries component that is known to exist, but is not recorded officially [57]. For example, an illegal trawl fishery, while its catch may be unknown, cannot have a catch of zero. Instead, we make the reasonable assumption that illegal foreign fleets, even where they are subsidised, have to recover a large fraction of the variable cost of their fishing operations, thus providing a baseline enabling the estimation of a minimum catch [6]. Catches reconstructed on this basis therefore include legal (but unreported) and illegal (and unreported) components by fleets from different parts of the world. Herein, we define legal catches as those obtained under legal fishing agreements regardless of adherence to host countries’ fishing regulations. These include catches by licensed foreign vessels fishing in prohibited artisanal fishing areas, using illegal gear, etc., as these catches are accounted for in legal catch estimations and are most likely a matter of domestic regulation violations rather than international crimes [58,59].

Official (reported) catch data by DWFs as reported to and by FAO cover the entire Eastern Central Atlantic (FAO area 34) and the Southeastern Atlantic (FAO area 47), and do not refer to specific EEZs. Therefore, to be able to compare the reconstructed catch data to official reported data, we used the catch data allocation approach developed by the *Sea Around Us* [60], which allocated catches to each EEZ in the FAO area studied from 1950 to 2006, updated to 2010 assuming the same spatial allocation for reported catches, i.e., proportionality. This approach allows to allocate landing statistics pertaining to huge FAO statistical areas, to EEZs and thus obtain informative catch distributions. On the other hand, proportionality assumes that unreported catches are correlated with reported catches meaning that the catch of the fleet within a certain EEZ is high, the higher their reported catch will be. The weakness of this approach consists in its inability to address the differences in fleet behavior as it assumes that all fleets behave similarly. We excluded Namibia as Namibian catches were considered domestic after independence. We then compared these reported catches to the reconstructed catches, and analyzed how Chinese and EU-West Africa agreements were performing.

### 2. Landed value of West African fisheries operated by the EU and China

Landed value is defined herein as the product of the average ex-vessel price and landings (including reported, unreported and illegal landings), and is computed using West African ex-vessel fish prices (see below). Landed value in West Africa is thus an underestimate of potential catch value, as it disregards the potential value of discards by foreign fleets, whose discards usually consist of species that would be marketable on West African markets. Herein, by-catch landed by DWF, usually flagged to West African countries, are included in the analysis.

To derive the landed value of the catch taken by EU and Chinese fleets, we used reconstructed catch data by country (Table 1), and average ex-vessel prices (in 2005 USD per tonne) extracted from Swartz et al. [61] for the period 2000 to 2010. We note that large differences in ex-vessel value exist, in some cases between neighboring countries, (e.g., hakes taken from Morocco are valued at $4000 US·t-1 in contrast to $118 US·t-1 in Mauritania [61]. We used the World Bank’s Consumer Price Index (CPI) per country to convert real prices to actual 2013 USD per tonne [2013 Price = [2000 to 2010] Price x (2013 CPI/2005 CPI)]. Using ex-vessel prices observed in regional markets to compute the landed value allows comparisons of landed values by foreign countries regardless of price differences due to fish demand and supply on the international (global) market. The landed value is thus obtained as the product of the average ex-vessel prices in 2013 USD times the corresponding reconstructed catch in each EEZ. We conducted the same analysis for EU and Chinese catches (Table 1).

### 3. Agreement value and landed value: How the EU compares to China

For a first comparison of landed value of the catch to the compensation paid by the EU and China under increasing competition to access the West African fishing grounds, we derived the average annual amount received by each of the West African countries listed above (Table 1) for 2010 or at the time of the latest fishing agreement prior to 2010.

European FPA data were available from the European Union law database (http://eur-lex.europa.eu). The amounts paid by the EU to access West African fisheries under FPAs (in Euro and including licence fees and other fees) were collected from the EU law database, converted into USD using the annual Euro to USD exchange rate for 2010, and then expressed in 2013 USD using the Consumer Price Index (CPI). Whether these are linked directly or indirectly to other trade arrangement is not considered here to be relevant as long as it is not clearly stated in the agreement itself.

In contrast to the FPAs of the EU which involve governments rather than companies, Chinese-West African agreements often include joint ventures and reflagged vessels even when the agreements involve government entities. The Chinese agreements considered here are those that fit this condition, i.e., they were inter-governmental agreement, rather than agreements between companies, and thus they do not fit the second generation agreement definition.

Although some China-West Africa agreements were documented, the amounts involved are often confidential or expressed in terms of a ‘project value’ provided in exchange for fishing access (e.g., construction of an official buildings in Gabon, provision of military jets in Mauritania, etc.). We consider the value of these projects as the value of the agreement itself when there is direct evidence that the project occurred in exchange for fishing access for the Chinese fleet, rather than as part of a package offered by China for several sectors including fisheries. Here, the value of such projects was assessed based largely on media reports and economic news (see S1 Table for examples and S1 Materials for a comprehensive account). One transparent example was the 150 million USD [62] that China paid to Morocco between 1988 and 2002 for all fishing access agreements and for joint ventures (Table 2), involving 26 companies (http://ma.china-embassy.org/fra/xwdt/t464813.htm). In contrast, Chinese fees paid to Mauritania were too opaque to be identified, other than in the very recent Mauritania-China Agreement (Table 2), with a compensation of 100 million USD for 25 years [63, 64]. The low transparency of Chinese fisheries agreements may have introduced a bias in the assessment conducted here. Hopefully, future Chinese agreements (or more detailed studies) will shed more light on this issue. In the meantime, we present our tentative result in the hope that it will contribute to more transparency in the future.

**Table 2:** Examples of computations of amount of Chinese payments to West African countries in exchange for fishing access.

Item	Country	Methods and references
Port infrastructure	Mauritania	Mauritania received a payment of 282 million USD [93]. We assumed construction period of 4 years, within which the project had to be delivered, the result was then converted to 2013 USD using CPI.
Construction of a dam and stadium*	Cape Verde	China funded the construction of a dam and a stadium, whose value were estimated as Euro 3.5 million and 12 million USD, as assessed by Escobar and Kimbamba Simões [94]. We assumed a construction period of 7 years, i.e., the duration of the project, and thus divided the amount by 7 and converted the result to USD assuming an exchange rate of 1 Euro = 1.33 USD. Then we converted the resulting value to 2013 USD.
Fishing ropes and net processing	Ghana	China paid 9.1 million USD for the project for one year [95]. We converted this value to 2013 USD using the appropriate CPI.
Construction of national assembly and senate	Gabon	China invested in the construction of the national assembly building (73 million USD) and the senate (1.2 million USD) in 2004 [96]. We converted these amounts to 2013 USD using the CPI.

*We found no document stating explicitly that these projects were to compensate for fishing access. However, the Chinese National Fishing Company (CNFC) was directly involved in these projects and, outside of fisheries, we know of no Chinese involvement in the economy of Cape Verde.

We compare the financial compensation paid by the EU and China for fishing access to the landed value of reported catches which we herein call the "official compensation rate", and to the total landed value by EU member countries and China based on reconstructed catches (“actual compensation rate”). We then estimated the difference between these two compensation rates as the minimum economic loss for West African countries. Therefore, to conduct this exercise, two conditions needed to be present: first, both EU and China had to have an agreement with the country under consideration; second, the amount paid for access (as defined above) had to be documented in some manner (real value, project value or anecdotal account). In the absence of a fishing agreement, catches (that are not under joint ventures or those considered domestic because of reflagging practices by the EU), and therefore their value, are considered illegal. This allowed quantifying the fraction of the landed value that was covered by fishing agreements by both Europe and China.

## Results

### 1. Catches by Europe and China

Our bottom-up estimation of Chinese catches from West Africa suggested that they caught on average 2.3 million t·year^-1^ from 2000 to 2010, which is around 20% lower than the midpoint estimate provided by Pauly et al. [6] for the West African region (i.e., 2.9 million t·year^-1^), but well within its 95% confidence interval, which ranged from 1.84 to 4.30 million t·year^-1^. Namibia (EEZ area 12% of total EEZ area) was not included in this analysis for the 2000s, which would further reduce the gap between the present estimate and the midpoint estimate by Pauly et al. [6]. This is comforting, given that these two estimates were obtained using radically different methods.

**Fig 2:**
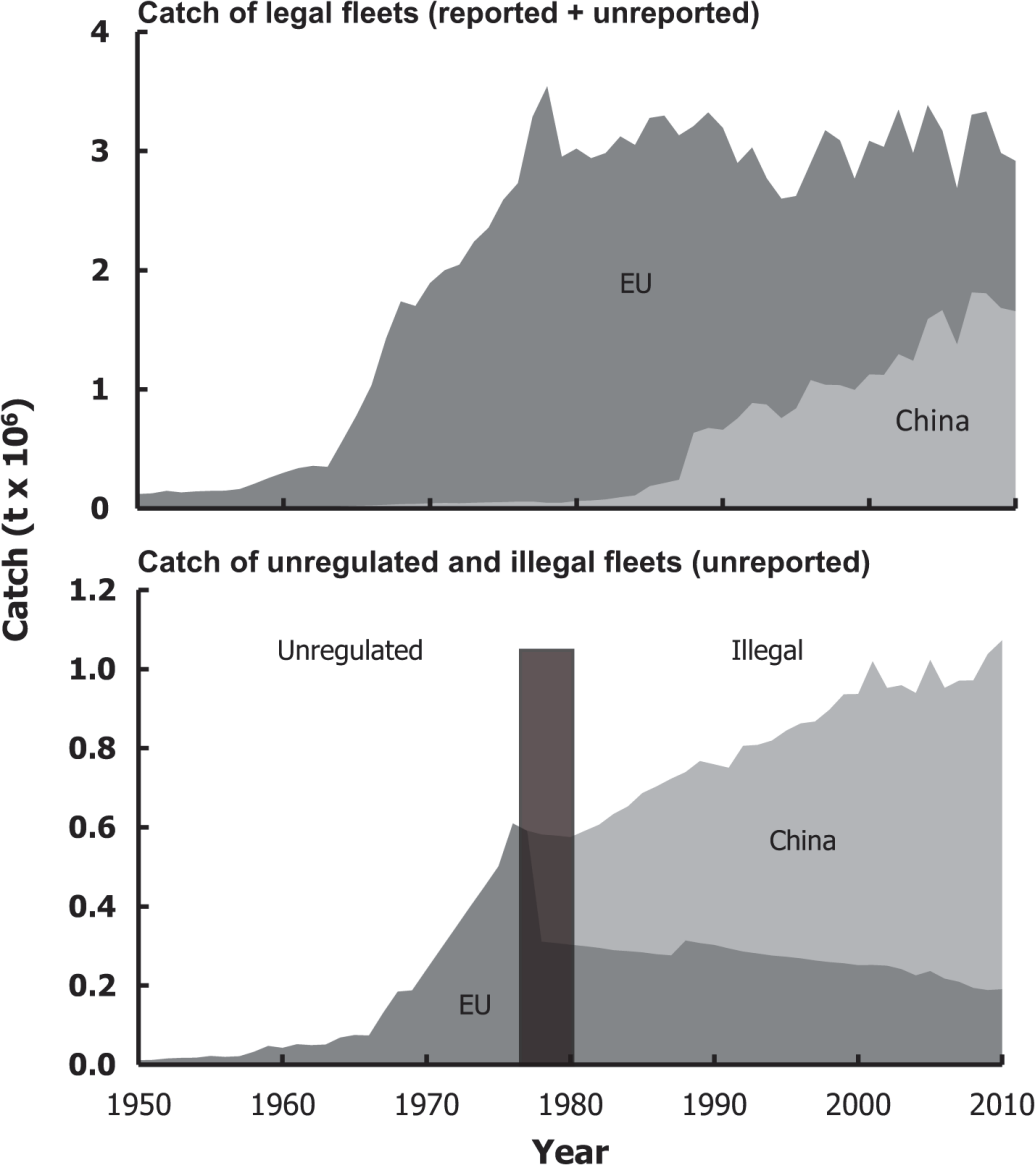
Total catches by Chinese and EU fleets from the waters of West Africa, 1950–2010. Sierra Lone claimed its EEZ in 1971, Morocco in 1981, Equatorial Guinea and Gabon in 1984, Congo (ex-Zaire) in 1992 and Cameroon in 2000. The 16 other West African countries claimed their EEZ between 1976 and 1980, and hence the location of the transition zone (shaded) from ‘unregulated; to ‘illegal’ catches in the lower panel.

Catches by Chinese fleets from West Africa increased rapidly after the mid-1980s and were estimated to include 1.4 million t·year^-1^ of legal, but unreported catches, 761,000 t·year^-1^ of illegal (or unregulated prior to EEZ declaration) and unreported catches, 20,200 t·year^-1^ of catches reported to the FAO (FAO data for FAO area 34 and 47 for China) and 159,000 t·year^-1^ of catches reported as domestic catches by West African fleets, but whose beneficial ownership was Chinese. Thus, only 8% of total catches were reported officially as being caught by China, which is the same as the estimate by Pauly *et al*. (2013). Under-reporting by China’s legal fleet was at its highest in the 1980s, when China began distant-water fishing operations in West Africa. Under-reporting by China decreased over time with increasing reflagging to West African countries as their catches were reported as domestic, but remained relatively high, as total legal catches were estimated to be, on average, 11 times higher than reported catches between 2000 and 2010 (Table 1). Illegal catches by Chinese fleets were estimated at over 357,000 t·year^-1^ on average during the 1980s, and increased gradually to around 761,000 t·year^-1^ on average during the 2000–2010 time period (Fig. 2).

Catches by legal EU fleets in West Africa were estimated at 155,000 t·year^-1^ in the 1950s, peaked at 3.5 million t·year^-1^ in the mid-1970s and declined thereafter to 1.8 million t·year^-1^ on average over the period from 2000 to 2010 (Fig. 2), of which only 524,000 t·year^-1^ were officially reported to the FAO, i.e., 30%. Catches by illegal (or unregulated prior to EEZ declaration) EU fleets, on the other hand, were estimated at 92,000 t·year^-1^ during the 1960s, increased to around 404,000 t·year^-1^ on average during the 1970s, were equivalent to Chinese illegal catches during the early 1980s and then decreased to around 224,000 t·year^-1^ on average during the 2000–2010 time period (Fig. 2).

European legal catches were relatively prominent, with on average 3.2 million t·year^-1^ during the 1980s, before China entered the West African fishing grounds, and were equivalent to catches by Chinese legal fleets during the early 2000s (Fig. 2). The decrease of EU catches from West African waters over time, in contrast to increasing Chinese catches over the 2000–2010 time period would suggest a gradual replacement of European fleets by Chinese fleets. However, another, more realistic scenario would explain the decrease in European contribution to foreign catches by the growth of reflagging practices by EU fleets to West African countries, e.g., Senegal [43]. Chinese reflagging practices were more readily unveiled, while the nature of the post-colonial relations between EU member countries and some West African countries made it difficult to distinguish between such reflagged fleets and truly domestic fleets.

### 2. Landed value of West African fisheries

Over the 2000–2010 time period, catches worth 8.3 billion USD were taken by EU (total value: 3.7 billion USD) and Chinese (total value: 4.7 billion USD) fleets operating in West Africa (Table 3). Most of this value (and the corresponding catch) originated from Morocco (see Table 1 for catch estimates) and notably from its southern part, the former Spanish Sahara (68%). Of the total value of 3.7 billion USD taken by the EU, 4% was taken illegally (landed illegal value: 0.2 billion USD), while 1.7 billion USD of the 4.7 billion USD taken by Chinese vessel was deemed illegal (i.e., 40%). Over 90% of the landed value by legal EU fleets was taken from Morocco, Mauritania, Cape Verde and Guinea, while the landed value taken by illegal EU fleets was mainly from Mauritania, Senegal and Liberia (Table 3). Similarly, most (over 96%) of the legal landed value by China was taken from the waters of Morocco (including those of Western Sahara), Mauritania, Angola, Cape Verde and the Congo (Brazzaville). Most (87% of illegal landed value) of the illegal fishing activities by China seem to be concentrated around Morocco, Nigeria and to a lesser extent Mauritania (Table 3).

**Table 3:** Estimated average landed value, access value, official and actual compensation by Europe and China from West African countries for the period between 2000 and 2010, in 2013 USD x 106.

	EU landed value(USD_2013_ x 10^6^)		China landed value(USD_2013_ x 10^6^)		Total value (USD_2013_ x 10^6^)		Access value (USD_2013_ x 10^6^)		Value of reported landings(USD_2013_ x 10^6^)		Official compensation (%)		Actual compensation (%)	
	Legal	Illegal	Legal	Illegal	EU	China	EU	China	EU	China	EU	China	EU	China
Morocco	2,592.6	0.0	1,758.9	1,238.6	2,592.6	2,997.5	180.3	9.2	844.9	212.0	21.0	4.0	7.0	0.3
Mauritania	295.7	115.7	508.2	54.7	411.5	562.9	95.2	102.0	95.3	122.1	100.0	83.0	23.1	18.1
Senegal	50.9	10.0	5.8	28.3	60.9	34.1	11.9	0.7	19.2	0.8	62.0	91.0	19.5	2.0
The Gambia	22.3	0.0	6.9	3.9	22.3	10.7	0.2	^a^	7.4	0.9	3.0		0.9	
Cape Verde	277.4	0.0	175.7	32.7	277.4	208.4	0.3	2.0	91.6	21.2	0.3	9.0	0.1	1.0
Guinea Bissau	25.4	2.2	23.9	10.0	27.6	34.0	5.7	2.9	8.6	3.0	66.0	97.0	20.5	8.5
Guinea	157.4	1.3	10.9	11.8	158.7	22.7	1.0	1.3	49.2	1.3	2.0	95.0	0.6	5.6
Sierra Leone	28.0	0.0	2.2	27.6	28.0	29.8	0.0	0.2	8.8	0.5		50.0	0.0	0.8
Liberia	17.8	19.1	9.1	21.6	36.9	30.6	0.0	^a^	5.9	1.0			0.0	
Côte d'Ivoire	4.3	0.0	16.3	126.8	4.3	143.1	0.4	0.7	1.4	1.9	31.0	36.0	10.3	0.5
Ghana	0.0	1.6	12.7	1.6	1.6	14.3	0.0	11.2	0.0	13.7		82.0	0.0	78.2
Togo	4.6	0.0	1.9	5.3	4.6	7.3	0.0	^a^	1.5	0.2	0.0	83.6	0.0	2.5
Benin	0.3	0.0	0.6	0.0	0.3	0.6	0.0	^a^	0.1	0.1	0.0		0.0	
Nigeria	0.0	0.0	0.0	160.9	0.0	160.9		^a^	0.0	0.0				
Cameroon	0.0	0.0	2.6	14.1	0.0	16.7	0.0	10.8	0.0	0.3		0.3		64.7
Equatorial Guinea	20.3	0.0	11.2	1.8	20.3	12.9	0.2	^a^	7.5	1.3	3.0		1.0	
Gabon	8.6	0.0	13.4	5.8	8.6	19.2	0.7	10.3	2.8	10.6	24.0	97.0	7.9	53.4
Sao Tome & Prin.	5.5	0.0	0.2	0.0	5.5	0.2	0.5	^a^	1.8	0.0	27.0		8.6	
Congo (Brazzaville)	0.0	0.0	98.0	36.9	0.0	134.9		^a^	0.0	10.4				
Congo (ex-Zaire)	0.0	0.0	1.3	0.3	0.0	1.6		^a^	0.0	0.2				
Angola	37.9	1.8	250.5	31.2	39.7	281.6	10.9	25.3	20.8	23.2	52.0	108.8	27.0	9.0
Namibia	16.4	0.0	0.0	0.0	16.4	0.0	0.0	0.0	0.0	0.0	0.0		0.0	
**Total value**	**3,549.1**	**151.8**	**2,910.0**	**1,814.0**	**3,717.2**	**4,724.0**	**307.2**	**176.7**	**1,166.8**	**424.7**	-	-	-	-
**Adjusted value** ^**b**^	3,549.1	151.8	**2,780.8**	**1,583.3**	3,717.2	**4,391.1**	307.2	176.7	1,166.8	**410.6**	26.0	**40.0**	**8.0**	**4.0**

a Evidence of an agreement was found, but its value could be not be estimated;

b sum of values for countries that have both catch and compensation values.

### 3. Agreements value and landed value: How Europe compares to China

European fishing agreements and equity

Europe paid on average 307 million USD·year^-1^ for access to West African waters from 2000 to 2010 (Table 3). The highest cumulative access fees were paid to Morocco and Mauritania with 180 million USD·year^-1^ and 95 million USD·year^-1^, respectively, followed by Senegal and Angola. Official compensation by the EU, calculated as the ratio between access value and the value of reported landings, was estimated at 26% (Table 3). This means that the EU pays back around a quarter of the value EU fleets report as taken from West Africa, which appears to be reasonable, particularly in the case of Mauritania which appears to receive an astonishing 100% of the reported landed value by EU fleets (Table 3). However, when the compensation is calculated as the ratio between access value and total landed value, including illegally caught fish (see Table 1), it is much lower at, on average 8% (Table 3). This value ranges between 0% (in countries where agreements were absent, but catches were documented, i.e., Benin, Sierra Leone, Togo, Liberia and Ghana) to a maximum of 23% (Mauritania, Table 3).

Similarly, although the financial compensation paid by the EU under the EU-Morocco FPAs is fairly high (180 million USD, Table 3) compared to other countries in the area, this represented only 7% of the actual landed value of total catches (Table 3). In terms of regional disparity, Morocco for example represents 28% of the total in catches and 59% in agreement fees, while Mauritania with a third of total catches, received only 30% of the agreement fees. Thus, the EU pays to Morocco around 345 USD·t^-1^, while it pays 154 USD·t^-1^ to Mauritania.

Chinese fishing agreements and equity

The estimation of access fees paid by China required thorough investigations in which documents such as media and news reports were given particular attention (see S1 Table and S1 Materials for a comprehensive account). Due to the poor transparency of Chinese agreements, we were unable to spot all cases of Chinese payments, even when there was evidence of an agreement (Table 3). We were also unable to fully separate the sums paid for sectors other than fisheries, which would cause an upward bias. We can only hope that these two biases compensate for each other (but see below).

The access fees paid by China (S1 Results and Discussion) were classified under three distinct categories:

- Direct monetary payments: China paid on average 1.3 million USD·year^-1^ to Guinea, 9 million USD·year^-1^ to Morocco (S1 Table). Another example is illustrated by the agreement between China and Mauritania for a total value of 100 million USD in 2010 for a period of 25 years. However, this value was not taken into consideration since China did not respect all terms of the agreement [65]. In this category, there is robust evidence suggesting that fees are paid exclusively in exchange of fishing access, and do not include other sectors.
- Licence fees: In most cases, Chinese fleets reflagged to West African countries pay lower fees. Sometimes these fees are comparable to the fees paid by truly domestic fleets. Our investigation revealed this to be the only method of payment for The Gambia (for which monetary values were not available, however), Sierra Leone for which a value of 0.2 million USD·year^-1^ was derived (see S1 Table) and Senegal, for which we estimated a total access value of 0.7 million USD·year^-1^. This method represented only part of the total payments for Mauritania, Guinea Bissau, Gabon and Côte d’Ivoire. There is also strong evidence that fees under this category were paid exclusively in exchange for fishing access and do not involve other sectors. In addition, the fact that this method was the only payment documented for some countries (Senegal and The Gambia) might have introduced a downward bias (when compared to EU FPAs).
- Indirect payments (e.g., infrastructure development, military equipment, debt relief, interest free loans and international donations): This method illustrates the practical nature of Chinese access fees as perceived by African countries [66], as they tend to contribute directly to the development of various sectors. Examples include a) investments in infrastructure, as port construction and extension (the ‘Quay of Friendship’ extension in Mauritania, reconstruction of fishing harbors in Guinea Bissau, Côte d’Ivoire and Ghana), social and health infrastructures, including hospitals (Mauritania, Cape Verde), telecommunication network modernizations (Guinea Bissau), sports stadium (Cape Verde) and other infrastructure projects such as Parliament and other buildings (Gabon and Guinea Bissau); b) military aid, e.g., to Mauritania, along with 2 military jets and other items to boost the Mauritanian defense forces; and c) debt relief (Cape Verde) and interest free loans (Côte d’Ivoire) (see S1 Table). These payments are rarely reported and documented officially, and can be inferred only through grey literature and news reports. It is also difficult to apportion a fraction directly related to fishing access, as some of these payments may be for other socio-political or economic reasons not specified by China. However, in most cases, there is evidence pointing at fishing access being provided in exchange for these payments.

We estimated that Chinese agreements with West African countries provided 166 million USD·year^-1^ on average as access fees from 2000 to 2010 (Table 3). However, not all access fees by China were available, given the highly non-transparent nature of Chinese fishing agreements [67]. The official compensation rate by China is estimated at 40% compared to an actual compensation rate of 4% when including illegally caught fish and adjusting the value to remove those countries for which access fees could not be documented (see ‘Adjusted values’ in Table 3). Thus, the actual compensation rate by China (4%) is half the actual compensation rate by the EU (8%) to West African countries (Table 3). A strong regional variation in the agreement value over the same species exists. Indeed, while the agreement amount with Morocco represented 5% of the total, Chinese catches from Morocco represented 26%, which translates into 15 USD·t^-1^ compared to 121 USD·t^-1^ paid to Mauritania.

Finally, to deal with the bias issue alluded above, more stringent criteria are applied for Chinese projects that are fisheries related. In such case, when projects equivalent to 15 million USD·year^-1^ that might not be exclusively related to fisheries are excluded (e.g., 0.67 million for Côte d’Ivoire, 2 million for Cape Verde, 2.74 million for Guinea Bissau and 4 million for Ghana), the 4% Chinese compensation would decline to 3%. Calculation methods described above using these numbers yielded a total of 15 million USD·year^-1^.

Overall, however, the huge variance around the mean compensation rates estimated for the EU and China imply that they are not statistically different (t-test, p = 0.05).

## Discussion

The present study shows that the EU increasingly under-reports catches from West African waters, while Chinese under-reporting, although higher, may be declining, which may also highlight the increasing trend toward the reflagging of Chinese fleets. This reflagging, in turn, resulted in a relatively higher compensation rate by the EU as “domestic vessels” usually pay lower fees.

Under-reporting directly impacts local economies and sustainability when it hides over-fishing. For example, European fleets increased their catches from Morocco and Western Sahara by 5% after 1995 despite the agreement by Europe and Morocco to decrease the EU fishing quota by 40% [54]. Besides, by threatening the long-term sustainability of fish stocks, foreign fleets preclude the development of domestic fisheries [68]. This has led Mauritania, for example, to exclude octopus from the newly signed EU-Mauritania FPA [69] now supposedly only accessed by the strictly ‘domestic’ fishery, which actually consists of reflagged Chinese and European vessels [28,70–72]. Moreover, under-evaluated fisheries catches combined with weak non-collective bargaining power and secrecy around the negotiation of fisheries agreements resulted in major financial arrangement discrepancies [68], particularly in the case of China, with reflagging to domestic fleets contributing to minimizing licence fees.

Landed value taken by EU and Chinese fleets from West African waters estimated at 8.4 billion USD·year^-1^ exclude added value (processing, marketing, etc.), which would increase the landed value by about 40% [7,70]. This would raise the annual value to $11.8 billion US·year^-1^, equivalent to the average net (total) development assistance and official aid received from all countries by the West African countries considered here from 2000 to 2010 (http://www.worldbank.org). The difference between landed values estimated here and what is paid to West African countries reflect a small proportion of the total loss experienced by African countries. However, they are probably a reasonable approximation of the realistic gross revenues generated by West African legal fisheries (excluding Namibia). West African governments earned about $0.5 billion, or 6%, the latter however excludes the value of local landing and processing activities and employment, which is relatively low.

Other components not discussed in the present study worsen the situation. Bribing by foreign companies to access local resources, for example, leads to abuses of ecological resources and human rights, as the more these countries depend on natural resources for their exports, the more corrupt they would be in selling these resources [27]. Sustainability-wise, the difference between domestic catches (i.e., official landings excluding non-commercial sectors and discards) and the potentially sustainable catch justifies agreements under the UNCLOS. However, concerns are raised when components other than landings are accounted for and this surplus exceeded. Also, under the terms of UNCLOS, fishing access agreements should not jeopardize local development and livelihoods.

If we assume that tangible projects (e.g., port developments) are more likely (at least in high-corruption countries) to have more of a positive impact on the economy than cash (which can easily be misappropriated), the Chinese approach to compensating West African countries for access to their fisheries resources appears strategic. However, both Chinese and EU fishing agreements have negative effects on the food security of the host countries [7,11,24,54].

The pattern of Europe paying relatively low fees to developing countries for their fisheries resources, confirmed in this contribution, was documented earlier for African countries, e.g., Madagascar and the Seychelles [7,8,38]; in fact our estimate of 8% was relatively high when compared to earlier estimates. On the other hand, although Chinese projects offered *in lieu* of cash may appear more attracting for highly corrupt countries, their amount being lower than the fees paid by the EU might be related to higher under-reporting, and the fact that the total value of each agreement might not be entirely covered by the present study. Moreover, there are strong regional variations where China has a higher compensation rate than the EU (Cape Verde, Guinea, Sierra Leone, Ghana and Gabon), which make the overall compensation rates statistically similar. However, we think that pending more detailed studies, our work suggests that both compensation rates by the EU and China are low.

While Chinese fishing agreements and joint venture practices with host countries are often opaque, China appears to perform similarly to the European countries in terms of under-reporting and illegal practices. As a result of this foreign fishing, stocks are over-exploited, local communities struggle to meet their income and nutritional needs [9], and fisheries suffer from imported over-capacity and over-exploitation. These conditions led Senegalese artisanal *pirogues* to go further afield and fish illegally in Mauritanian waters [43], Guinean fishers to increase their costs of fishing, and the Mauritanian government to enforce a closed octopus fishing season to artisanal fishers [73]. These negative impacts are not only limited to the socio-economic behavior of fishers but have also disastrous consequences on fish stocks like the decrease in average size of fish, decrease in catch per unit of effort of various species [74,75] and in the long run ‘importing’ depletion of marine stocks rather than exporting fisheries resources [8,26].

While Chinese agreements with West African countries may be strategic in term of economic development, they never include financial contributions to support monitoring and surveillance and scientific research used, and these items usually fall by the wayside. The EU, which insists on monitoring and surveillance, on the other hand, underpays the fees related to these crucial activities [7], as illustrated by the very high presence of illegal vessels in the West African EEZs, including EU vessels. Moreover, EU fleet owners, like most foreign fleet owners, do not allow host country observers onboard. The lack of these components, being of crucial importance to enforcing fishing limits for example, contributes to increasing illegal fishing activities. Although Chinese fleets are increasingly responsible for a host of illegal, unreported and unregulated practices in West Africa, including unlicensed fishing, fishing in artisanal areas, use of illegal fishing gear, trans-shipping etc., EU operators are also responsible for relatively high illegal catches.

Our recommendations to improve the fairness, equity and transparency of the access agreements on the one hand and fisheries sustainability on the other hand are few. First, one can apply the principle of *Monitoring the Monitor* [76], to ensure monitoring fees are used appropriately. Second, increased transparency of agreements and intra-government communications may lead to competition between DWF countries, thus higher fees paid under agreements which will likely improve the negotiating position of West African countries by increasing financial competition for the same resources. Therefore, negotiating regional agreements, by increasing the collective bargaining power of West African countries, could yield better results in terms of value and sustainability, compared to the present situation. Moreover, West African countries must carry out regulatory responsibilities at a regional level and enforce MSC agreements already in place such as the right of pursuit. Establishing a registry of reflagging and flag of convenience vessels that is easily accessible will also result in higher transparency at local and regional levels. Moreover, as present GRT measures used as resource allocation criteria are ineffective, more appropriate allocation measures should be in place such as species quota allocation. Funds within the fishing access agreements should be clearly dedicated towards the establishment and enforcement of such criteria and the support of scientific research and training.

While the EU has prioritized the rebuilding of fish stocks within its waters, increasing DWF capacity has resulted in overfishing in West African countries. Both the EU and China should act with more responsibility with regards to the fisheries they endorse in the West African region. For example, sanctions should be implemented against vessels denying access to observers onboard or fishing above quotas. This adds to efforts of effective controls over fleet activity by enforcing measures already in place under fishing agreements (e.g., non-use of destructive gear, quota and by-catch limitations). Similarly, some effort should be directed towards capacity building in African countries particularly to enhance local MCS capacity.

On a development perspective, African countries should prioritize (foreign) access to resources to operators that offload their catches in local ports to allow for added value through processing and marketing operations.

Besides the bias that may have been introduced to the present assessment by the low transparency of Chinese fishing agreements, weaknesses of the present study consist in its inability to further document conditions under which fishing agreements were negotiated, may they be economic or political. These, although usually confidential, play a major role in determining access and the underlying access fees. Moreover, some access fees paid by China were excluded from the present assessment due to the failure to separate out the component that was strictly reserved for fishing access and the bribes offered by fishing companies. This may have contributed to under-estimating the amount paid by China for fishing access. Further investigation of these fees is needed to eliminate such bias. Further research may compare other non-monetary aspects of fishing agreements not included here, such as their contribution to supporting MCS and scientific research and training and the inclusion of sustainability clauses and their implementation. Besides the bias that may have been introduced to the present assessment by the low transparency of Chinese fishing agreements, weaknesses of the present study consist in its inability to further document conditions under which fishing agreements were negotiated, may they be economic or political. These, although usually confidential, play a major role in determining access and the underlying access fees. Moreover, some access fees paid by China were excluded from the present assessment due to the failure to separate out the component that was strictly reserved for fishing access. This may have contributed to under-estimating the amount paid by China for fishing access. Further investigation of these fees is needed to eliminate such bias. Further research may compare other non-monetary aspects of fishing agreements not included here, such as their contribution to supporting MCS and scientific research and training and the inclusion of sustainability clauses and their implementation.

## Supporting Information

S1 Materials Materials and methods for estimating the annual value of Chinese legal access to West African fishing grounds, 2000–2010. (DOCX)

S1 References Other references used in S1 Table for the assessment of the fees paid by China for access to the EEZs of West African countries. (DOCX)

S1 Results and Discussion Results and main findings summarized from the assessment presented in S1 Table. (DOCX)

S1 Table Materials for estimating the annual value of Chinese legal access to West African fishing grounds, 2000–2010. (DOCX)
