# Supplementary material for: Euros vs. Yuan: Comparing European and Chinese Fishing Access in West Africa
Source: PLoS One. 2015 Mar 20;10(3):e0118351. doi: 10.1371/journal.pone.0118351 (PMC4368511; doi:10.1371/journal.pone.0118351)
Supplement: S1 Materials — (DOCX) [file pone.0118351.s001.docx]

**S1 Materials** Materials and methods for estimating the annual value of Chinese legal access to West African fishing grounds, 2000-2010

These supplementary materials present the compilation of documents presenting evidence of the presence of legal access (agreement) by the People Republic of China to the Exclusive Economic Zones of West African countries, and the value/fees paid under these agreements, as reported in the scientific literature, in the mass media and on the websites of governmental and non-governmental organizations. The data were all accessed between June and July 2014.

We investigated the legal presence of vessels from mainland China in 22 West African countries: Morocco, Mauritania, Senegal, The Gambia, Cape Verde, Guinea Bissau, Sierra Leone, Guinea, Liberia, Cote d’Ivoire, Ghana, Togo, Benin, Nigeria, Cameroon, Gabon, Equatorial Guinea, Congo, Congo Dem. Rep., Sao Tome and Principe, Angola and Namibia. Once evidence of legal access (e.g., joint ventures, inter-government agreements, vessel chartering etc.) was documented for a country, we investigated the value of such access in three steps:

First, we searched for the nature of the payment: Chinese payments were classified in three different categories:

a) Category I, Direct Monetary payments: China pays directly for the fishing agreement in which case the monetary value is documented and the duration of the agreement clearly stated. Examples include Guinea [1](Table 1) and Mauritania [2]. b) Category II, Licence fees: Chinese vessels pay licence fees, particularly when they are chartered or operated under joint ventures. These payments can be exclusive in which case they are the only payment offered by Chinese vessel operators to the West African country such as those in Senegal [3] and The Gambia (Government of The Gambia, unpub. data) (Table 1). Licence fee payments, in a few instances, are complementary to a larger investment in exchange for the presence of the Chinese fleet in the West Africa. Examples include Guinea Bissau, Mauritania, Côte d’Ivoire where the Chinese fleet paid licence fees in addition to an amount paid by China to access fishing grounds (Table 1), which usually are classified as a third category (indirect payments);

c) Category III, indirect payments: This method means that China pays for the legal access to West African fishing grounds by offering different ‘items’ in exchange. These category is relatively harder to investigate evidence has to demonstrate that the ‘payment’ is aimed in exchange for fisheries access, then the investigation has to reveal the nature of the item (infrastructure building, debt cancellation, military aid etc.), and the value of the item (Table 1). The payment is considered to be in exchange for fishing access whenever the reference mentions, establishes (or widely interprets as such) the direct and exclusive link between the payment (or the item) and the fishing access, or the fact that the item was directly provided by e.g. the Chinese National Fishing Company. If this mention was not available, we attempted to answer the question of whether or not fishing was the only (or the most important) economic activity of China in the host country (if sectors such as mining, telecommunications, etc. were not involved), e.g. Cape Verde, in which case there would be a strong link between fishing access and the amount paid, despite the possibility of the agreement including other sectors.

Second, once the nature of the payment was established, we searched for the items China paid for and their value, along with the duration of the project/agreement (Table 1).

Third, we converted local currencies and Euros to USD and USD_2013_ using the Consumer Price Index (CPI) per country ([www.worldbank.org](http://www.worldbank.org)) to convert values to USD_2013_ [2013 Price = [2000-2010] value x (2013 CPI/2005 CPI)].
