# Supplementary material for: Euros vs. Yuan: Comparing European and Chinese Fishing Access in West Africa
Source: PLoS One. 2015 Mar 20;10(3):e0118351. doi: 10.1371/journal.pone.0118351 (PMC4368511; doi:10.1371/journal.pone.0118351)
Supplement: S1 References — (DOCX) [file pone.0118351.s002.docx]

**S1 References** Other references used in Table S1 for the assessment of the fees paid by China for access to the EEZs of West African countries.

1. Sidibe A (2003) Les ressources halieutiques démersales côtières de la Guinée : exploitation, biologie et dynamique des principales espèces de la communauté à Sciaenidés. Thèse de Doctorat de l'ENSA de Rennes, Agrocampus Ouest. 320 p.

2. CFFA (2012) CFFA participation. FAO Committee on Fisheries - COFI 30 - Rome, 7 to 13 July 2012. Rome: Coalition for Fair Fisheries Arrangements. 31 p.

3. Anon. (2013) Arrêté Interministériel fixant les montants et les modalités de paiement des redevances des licences de pêche industrielle pour les navires battant pavillon sénégalais et les navires affrétés pour l'année 2013. Dakar: Ministère de la Pêche et des Affaires maritimes. 3 p.

4. Anon. (2002) China Welcomes Anything that Sells Well from Morocco. People Dayli August 26, 2002: 2.

5. Bureau of Economic and Business Affairs (2013) 2013 Investment Climate Statement - Mauritania. Washington, DC: U.S. Department of State.

6. Escobar A, Kimbamba Simões G (2012) Building on ambition: China helps build Cape Verde after independence. Macau: Macao Magazine.

7. Ahmad W, Amrani Joutey M, Sanfo O (2012) Accords Sino-Africans. Le développement des pays d’Afrique est-il susceptible de se réaliser en coopération avec la Chine plus qu’il ne l’a été avec la France? : ESSEC Business School 29 p.

8. Belhabib D (2014) Côte d’Ivoire: fisheries catch reconstruction, 1950-2010. In: Belhabib D, Pauly D, editors. Marine Fisheries Catches in West Africa, 1950-2010, Part II. Vancouver: Fisheries Centre, University of British Columbia.

9. African Centre for Economic Transformation (2009) Looking East: China-Africa engagements. Ghana country case study. Accra: African Centre for Economic Transformation. 33 p.

10. Glaesel Frontani H, McCracken A (2012) China's development intitiatives in Ghana, 1961-2011. Journal of Sustainable Development in Africa 14: 275-286.

11. Vogt J, Teka O, Sturm U (2010) Modern issues facing coastal management of the fishery industry: A study of the effects of globalization in coastal Benin on the traditional fishery community. Ocean & Coastal Management 53: 428-438.

12. Bricola P (2008) Développement du commerce des produits halieutiques. Brussels: Union Européenne. 130 p.

13. Bignouma G (2007) Coopération internationale et exploitation des ressources halieutiques au Gabon. Les Cahiers d’Outre-Mer 240: 1-10.

14. Anon. (2009) Synthèse du registre des licences de pêche pour les années 2008, 2009. Libreville: Ministère de l'agriculture, de l'élevage, de la pêche et du développement rural, Direction générale des pêches et de l'aquaculture. 5 p.

15. Ekouala L (2013) Le développement durable et le secteur des pêches et de l'aquaculture au Gabon : une étude de la gestion durable des ressources halieutiques et de leur écosystème dans les provinces de l’Estuaire et de l’Ogooué Maritime. Lille: Université du Littoral Côte d’Opale, Ecole doctorale SESAM (E.D n°73), Laboratoire T.V.E.S (E.A n°4477). 408 p.

16. Anon. (2010) Publication du registre des licences de pêches. Situation au 1er semestre 2010. Libreville: Ministère de l'agriculture, de l'élevage, de la pêche et du développement rural, Direction générale des pêches et de l'aquaculture. 4 p.

17. Anon. (2011) Liste des recettes générées par les licences de pêche 1er semestre 2011. Libreville: Ministère de l'agriculture, de l'élevage, de la pêche et du développement rural, Direction générale des pêches et de l'aquaculture. 3 p.

18. Oceanic Développement, Poseidon Aquatic Resource Management Ltd, MegaPesca Lda (2004) Framework contract for performing evaluations, Impact Analyses and Monitoring Services in the context of Fisheries Partnership Agreements concluded between the Community and Non-Member coastal states. Sao Tome and Principe. Concarneau, France: European Commission. 126 p.

19. Mavinga Ngembo J (2008) Rapport d’enquête sur l’impact des importations des poissons sur la pêche artisanale au bas-fleuve Kinshasa: Centre Régional d'Appui et de Formation pour le Développement. 15 p.

20. Du Preez M-L (2009) Fishing for Sustainable Livelihoods in Angola: The Co-operative Approach. Johannesburg: South African Institute of International Affairs. 27 p.

21. Campos I, Vines A (2008) Angola and China: a pragmatic partnership. Working Paper Presented at a CSIS Conference, “Prospects for Improving U.S.-China-Africa Cooperation,” December 5, 2007 Chatham House, London: Centre for Strategic and International studies. 26 p.

22. Immanuel S (2013) Namibia: Fishing Rights Holders' Shotgun Marriages in Stormy Waters. AllAfrica.
