# Supplementary material for: Euros vs. Yuan: Comparing European and Chinese Fishing Access in West Africa
Source: PLoS One. 2015 Mar 20;10(3):e0118351. doi: 10.1371/journal.pone.0118351 (PMC4368511; doi:10.1371/journal.pone.0118351)
Supplement: S1 Results and Discussion — (DOCX) [file pone.0118351.s003.docx]

**S1 Results and discussion** Results and main findings summarized from the assessment presented in S1 Table.

The literature review encompassed a wide range of sources and overall, over 33 items within the 22 countries investigated. As the value of 9 items could not be retraced, this represents a total coverage of 73%. Explicit evidence of a direct link of an investment to fisheries was taken as such, while implicit evidence or hints to a potential link was further investigated. For each country, we present the time period covered by the legal presence, the category of the payment, the item paid, the value of the payment converted to current $ US and the method and references.

Results show that the payment Category III (indirect payment) was the most common with 18 projects in 10 countries, followed by Category II (licence payments) paid to 8 countries and finally only one instance of direct fishing agreement payment (Table 1). In 50% of the cases, Category II is complementary to Category III.

While the payment for Cameroon was not taken into account later in the study, payments to Cape Verde (average annual amount 2 million USD), Guinea Bissau parliament reconstruction (2.74 million USD), Côte d’Ivoire EXIM BANK loan (0.67 million USD) and Ghana interest free loan (19 million USD) might also be directly related to other sectors such as mining. The inclusion of the above mentioned amounts of sectors other than fishing might have created an upward bias of 15 million USD when estimating annual averages.
