# Supplementary material for: Euros vs. Yuan: Comparing European and Chinese Fishing Access in West Africa
Source: PLoS One. 2015 Mar 20;10(3):e0118351. doi: 10.1371/journal.pone.0118351 (PMC4368511; doi:10.1371/journal.pone.0118351)
Supplement: S1 Table — (DOCX) [file pone.0118351.s004.docx]

| **S1 Table.** Materials for estimating the annual value of Chinese legal access to West African fishing grounds, 2000-2010. | | | | | | |
| --- | --- | --- | --- | --- | --- | --- |
| Country | Years | Category | Item | Cash value  (USD) | Cash value (cUSD_2013_·year^-1^∙10^6^) | Method |
| Morocco | 1988-2002 | III | Not mentioned | 150∙10^6^ | 9.24 | Total value of indirect payments provided [4] for the 1988-2002 in USD was divided by the number of years and then adjusted by the CPI. The average for the 2000s was taken assuming it was constant since then. |
| Mauritania | 1999 | II | Licence payment for total Chinese fleet | 170,443 | 0.21 | The license value was in USD (IMROP, unpub. data) was converted to USD_2013_ using CPI. |
| Mauritania | 2000 | II |  | 99,037 | 0.15 |  |
| Mauritania | 2001 | II |  | 40,000 | 0.04 |  |
| Mauritania | 2002 | II |  | 12,400 | 0.02 |  |
| Mauritania | 2006 | II |  | 229,531 | 0.18 |  |
| Mauritania | 2007 | II |  | 594,945 | 0.40 |  |
| Mauritania | 2008 | II |  | 661,050 | 0.44 |  |
| Mauritania | 2009 | II |  | 594,945 | 1.32 |  |
| Mauritania | 2010 | II |  | 944,370 | 0.74 |  |
| Mauritania | 2011 | II |  | 4,077,670 | 3.57 |  |
| Mauritania | 2012 | II |  | 3,697,350 | 3.70 |  |
| Mauritania | 2010 | III | Donation to boost defense^[[1]](#footnote-1)^ | 2∙10^6^ | 1.17 | This payment value in USD was for one year, the value in USD was converted to Current USD_2013_ using CPI |
| Mauritania | 2010 | III | Armed forces financial^[[2]](#footnote-2)^ support | 3∙10^6^ | 2.40 |  |
| Mauritania | 2010-2015 | III | Hospital, commercial centre and housing^[[3]](#footnote-3)^ | 100∙10^6^ | 13.10 | The total payment received in USD was divided by an assumed construction period of 6 years, within which the project had to be delivered, the result was converted to USD_2013_ using CPI |
| Mauritania | 2010-2013 | III | Port infrastructure | 282∙10^6^ | 55.40 | The total payment received in $ million US [5] was divided by an assumed construction period of 4 years, within which the project had to be delivered, the result was converted to USD_2013_ using CPI |
| Senegal | 2000-2010 | II | Licence payment per chartered tuna boat | 74,000 | 0.02 | The value per license was given in CFA (2013)/GRT/year for tuna vessels [3]. The number of Chinese tuna boats was given by Belhabib et al. 2014 (IUU) as 1 and the average GRT of Chinese boats was estimated at 308. The product of the GRT, the number of tuna boats and the license fee per boat in CFA, then converted to USD_2013_ using an exchange rate of 1 USD = 476 CFA |
| Senegal | 2000-2010 | II | Licence payment per chartered demersal boat | 37,000 | 0.67 | The value per license was given in CFA (2013)/GRT/year for demersal vessels [3]. The number of Chinese demersal boats was given by Belhabib et al. 2014 (IUU) as 14 and the average GRT of Chinese boats was estimated at 308. The product of the GRT, the number of demersal boats and the license fee per boat in CFA, then converted to USD_2013_ using an exchange rate of 1 USD = 476 CFA |
| The Gambia | 2004 | II | Licence payment for total Chinese fleet | Not found |  | There was evidence of licence based agreements and the details of 10 fishing vessels were available in the Gambian government unpublished data, no license fees were available |
| The Gambia | 2005 | II | Licence payment for total Chinese fleet | Not found |  | There was evidence of licence based agreements and the details of 5 fishing vessels were available in the Gambian government unpublished data, no license fees were available |
| Cape Verde | 2000-2006 | III | Dam building (3.5 million Euro) and Stadium building (12 million USD) | 17∙10^6^ | 3.00 | The value was documented in real USD [6]. We assumed a construction period of 7 years which is the duration of the project. We divided the amount by 7 and then converted it to USD assuming an exchange rate of 1 Euro = 1.33 USD and then converted the resulting value to Current USD |
| Cape Verde | 2006-2010 | III | Hospital building | 5∙10^6^ | 0.80 | The value was documented in real USD [6]. We assumed a construction period of 5 years, which is the initial duration of the project. We divided the total amount by 5, and then adjusted by the CPI to convert the values to USD_2013_. The value for 2006 takes into consideration the annual value for dam building, stadium building and hospital building. |
| Cape Verde | 2008 | III | Debt cancellation | 2∙10^6^ | 0.50 | The value was documented in real USD^[[4]](#footnote-4)^. This was assumed to be a one year payment, as no further evidence suggested otherwise |
| Guinea Bissau | 2007 | III | Reconstruction of parliament | 18∙10^6^ | 2.74 | The value was documented in USD^[[5]](#footnote-5)^. We assumed the duration of the project was one year. We converted USD to USD_2013_ using CPI. The reconstruction of the parliament was a fisheries related venture. |
| Guinea Bissau | 2007 | II | Licence payment for total Chinese fleet |  | 1.10 | Licence fees paid by China were 14% higher than those paid by the EU (223 Euros per GRT), i.e., 254 Euro/GRT^[[6]](#footnote-6)^. The Chinese fleet operating in Guinea Bissau had a total of 3,984 GRT. We estimated the total licence fees by multiplying the licence fees for China and then converted these to USD assuming an exchange rate of 1.33. We converted the resulting value to USD_2013_ using CPI. |
| Guinea | 1997-1998 | I | Direct monetary payments | 6 | 2.27 | The value was documented in USD[1]. The direct value of the agreement was converted to USD_2013_ using the CPI. |
| Guinea | 2001-2002 | I |  | 2 | 0.91 |  |
| Guinea | 2003-2004 | I |  | 2 | 0.61 |  |
| Sierra Leone | 2011 | II | Licence payment for total Chinese fleet | 376,500 | 0.24 | The value was documented in USD [7]. Over 50% of the demersal trawl fleet registered to Sierra Leone in the 2000s was allegedly of Chinese origin. Thus we divided licence revenues of trawlers by 2 and then converted the resulting value to USD_2013_ using CPI |
| Cote d'Ivoire | 2004 | II | License fee per boat | 3,076 | 0.02 | The value was documented in USD^[[7]](#footnote-7)^. There were 8 Chines vessels legally operating in Cote d'Ivoire per year [8]. We multiplied the latter number by the license fee then converted the resulting value to USD_2013_ using CPI. |
| Cote d'Ivoire | 2013 | III | EXIM BANK loan to Côte d'Ivoire | 321∙10^6^ | 0.67 | The CFA (2013) value provided^[[8]](#footnote-8)^ was converted to USD assuming an exchange rate of 1 USD = 472 CFA |
| Côte d'Ivoire | 2009 | III | Renovation of fishing port | Not found |  | This investment^[[9]](#footnote-9)^ was related to fisheries; however, its value was not was not found. The access value for Côte d'Ivoire is likely under-estimated |
| Ghana | 1998 | III | fishing ropes and net processing project | 9∙10^6^ | 7.00 | The value was documented in USD [9]. The project was over a one year period. We converted USD to USD_2013_ using CPI. |
| Ghana | 2008 | III | Construction of a fish pond (CNFC and Lu Ye Fisheries) in 2009 USD^[[10]](#footnote-10)^ | 40∙10^6^ | 7.00 | The value was documented in USD. The project was assumed to be over a 4 year time period. We divided the total amount by 4 then converted USD to USD_2013_ using CPI |
| Ghana | 2009 | III | interest-free loan to construct landing sites for fishing communities, provide social services, build schools and hospitals, and continue educational exchanges and scholarships [9,10] | 99∙10^6^ | 19.00 |  |
| Togo | 2004 | III | Not mentioned | 4∙10^6^ | 0.20 | The value was documented in USD^[[11]](#footnote-11)^. We assumed the investment was over a period of 5 years, as a conservative duration of Chinese agreements. We divided the original value by 5 and then converted USD to USD2013 using CPI |
| Benin | 2002-2010 |  | Not found | Not found |  | Evidence of agreements was found [11] but no value was available |
| Nigeria | 2004-2005 |  | Not found | Not found |  | Evidence of legal access^[[12]](#footnote-12)^, but the value could not be traced |
| Cameroon | 2013-undated | III | Donation for the development of fisheries | 60∙10^6^ |  | The value was documented in USD^[[13]](#footnote-13)^. The value for Cameroon was not taken into consideration as data on Chinese catches from the EEZ of Cameroon were not yet available. |
| Equatorial Guinea | 2005-2010 |  | Not found | Not found |  | Evidence of agreements was found [12], but no value was available |
| Gabon | 2004 | III | Construction of the national assembly building (73 million USD) and the senate (1.2 million USD) | 74∙10^6^ | 9.60 | The value was documented in real USD^[[14]](#footnote-14)^. We assumed these were a one year payment [13]. We converted USD to USD_2013_ using CPI. |
| Gabon | 2008 | II | License fee per boat | 17,176 | 0.30 | The license value in Euro (2013) applied to national and reflagged vessels. There were 13 Chinese boats reflagged to Gabon [14]. We obtained the total licence fee value as the product of licence fees per boat [15] for this category and the number of Chinese boats, converted to USD assuming an exchange rate of 1.33 |
| Gabon | 2008 | II |  | 51,525 | 1.78 | License value in Euro (2013) applied to foreign vessels [15]. There were 26 Chinese boats flagged to China and FoC [14]. The total licence fee value was computed as the product of licence fees per boat for this category and the number of Chinese boats, converted to USD, using an exchange rate of 1.33. |
| Gabon | 2009 | II |  | 17,176 | 0.27 | The license value in Euro (2013) applied to national and reflagged vessels [15]. There were 12 Chinese boats reflagged to Gabon [14]. We obtained the total licence fee value as the product of licence fees per boat for this category and the number of Chinese boats, converted to USD assuming an exchange rate of 1.33. |
| Gabon | 2009 | II |  | 51,525 | 0.96 | The license value in Euro (2013) applied to foreign vessels [15]. There were 14 Chinese boats flagged to China and FoC [14]. We obtained the total licence fee value as the product of licence fees per boat for this category and the number of Chinese boats, converted to USD assuming an exchange rate of 1.33. |
| Gabon | 2010 | II |  | 17,176 | 0.21 | The license value in Euro (2013) applied to national and reflagged vessels [15]. There were 9 Chinese boats reflagged to Gabon [16]. We obtained the total licence fee value as the product of licence fees per boat for this category and the number of Chinese boats, converted to USD assuming an exchange rate of 1.33. |
| Gabon | 2010 | II |  | 51,525 | 0.75 | The license value in Euro (2013) applied to foreign vessels [15]. There were 11 Chinese boats flagged to China and FoC [17]. We obtained the total licence fee value as the product of licence fees per boat for this category and the number of Chinese boats, converted to USD assuming an exchange rate of 1.33. |
| Gabon | 2011 | II |  | 17,176 | 0.25 | The license value in Euro (2013) applied to national and reflagged vessels [15]. There were 11 Chinese boats reflagged to Gabon [17]. We obtained the total licence fee value as the product of licence fees per boat for this category and the number of Chinese boats, converted to USD assuming an exchange rate of 1.33 |
| Gabon | 2011 | II |  | 51,525 | 0.75 | The license value in Euro (2013) applied to foreign vessels. There were 11 Chinese boats flagged to China and FoC. We obtained the total licence fee value as the product of licence fees per boat for this category and the number of Chinese boats, converted to USD assuming an exchange rate of 1.33 |
| Sao Tome and principe | 2000-2010 |  | Not found | Not found |  | Evidence of agreement [18] |
| Congo | 2013 | II | License fee per boat | Not found |  | Strong evidence of the presence of Chinese fleet fishing under license; however values and agreements could not be found. Even the text about the licences is not clear about the licence cost^[[15]](#footnote-15)^ |
| Congo, Dem. Rep. | 2000-2010 |  | Not found | Not found |  | Evidence of legal access, but value not found [19] |
| Angola | 2004-2010 | III | Building fishing infrastructure and artisanal and industrial fishing boats | 311 | 25.30 | The value was documented in USD [20,21]. We assumed the project lasted 7 years as it was still underway in 2008, and divided the total investment value by 7, then converted USD to USD_2013_. |
| Namibia |  |  |  |  |  | Evidence of joint ventures were found for 2013 with a value of N$ 24 million [22]; however no evidence was found for the period prior to 2013]. |

1. <http://china-defense.blogspot.ca/2010/04/china-donates-15-million-to-boost.html> [↑](#footnote-ref-1)
2. <http://sahelblog.wordpress.com/2011/09/20/mauritania-and-china/> [↑](#footnote-ref-2)
3. <http://www.wikileaks.org/plusd/cables/09NOUAKCHOTT236_a.html> [↑](#footnote-ref-3)
4. <http://www.capeverde.com/forum/cape-verde-general-forum-f2/china-s-relationship-with-cape-verde-t3169.html> [↑](#footnote-ref-4)
5. <http://en.m.wikipedia.org/wiki/China%E2%80%93Guinea-Bissau_relations> [↑](#footnote-ref-5)
6. <http://transparentsea.co/index.php?title=Guinea,Bissau:Industrial_fisheries#Fisheries_agreements_with_China> [↑](#footnote-ref-6)
7. <http://www.koffi.net/koffi/actualite/3129-Reportage-Peche-industrielle-Les-bateaux-chinois-coulent-les-armateurs-ivoiriens.htm> [↑](#footnote-ref-7)
8. <http://www.gouv.ci/actualite_1.php?recordID=3122> [↑](#footnote-ref-8)
9. <http://webcache.googleusercontent.com/search?q=cache:kW6w0lzs3KIJ:cabinetpkdconseil.com/a-lassaut-des-entreprises-chinoises330/&hl=fr&gl=ca&strip=1> [↑](#footnote-ref-9)
10. <http://china.aiddata.org/projects/1872> [↑](#footnote-ref-10)
11. <http://www.farmer.com.cn/wlb/yyb/yy7/200408310521.htm> [↑](#footnote-ref-11)
12. http://wenku.baidu.com/view/750b38303968011ca3009138.html?from=related&hasrec=1 [↑](#footnote-ref-12)
13. <http://www.investiraucameroun.com/tags/peche> [↑](#footnote-ref-13)
14. <http://www.agoravox.fr/actualites/international/article/la-chine-un-partenaire-economique-25872> [↑](#footnote-ref-14)
15. <http://faolex.fao.org/cgi-bin/faolex.exe?rec_id=000084&database=faolex&search_type=link&table=result&lang=fra&format_name=@FRALL> [↑](#footnote-ref-15)
